# A Fourier Transformation based Method to Mine Peptide Space for Antimicrobial Activity

**DOI:** 10.1186/1471-2105-7-S2-S2

**Published:** 2006-09-26

**Authors:** Vijayaraj Nagarajan, Navodit Kaushik, Beddhu Murali, Chaoyang Zhang, Sanyogita Lakhera, Mohamed O Elasri, Youping Deng

**Affiliations:** 1 Department of Biological Sciences, The University of Southern Mississippi, Hattiesburg, MS, 39406, USA; 2 Department of Computer Science, The University of Southern Mississippi, Hattiesburg, MS, 39406, USA; 3 Department of Mathematics, The University of Southern Mississippi, Hattiesburg, MS, 39406, USA

## Abstract

**Background:**

Naturally occurring antimicrobial peptides are currently being explored as potential candidate peptide drugs. Since antimicrobial peptides are part of the innate immune system of every living organism, it is possible to discover new candidate peptides using the available genomic and proteomic data. High throughput computational techniques could also be used to virtually scan the entire peptide space for discovering out new candidate antimicrobial peptides.

**Result:**

We have identified a unique indexing method based on biologically distinct characteristic features of known antimicrobial peptides. Analysis of the entries in the antimicrobial peptide databases, based on our indexing method, using Fourier transformation technique revealed a distinct peak in their power spectrum. We have developed a method to mine the genomic and proteomic data, for the presence of peptides with potential antimicrobial activity, by looking for this distinct peak. We also used the Euclidean metric to rank the potential antimicrobial peptides activity. We have parallelized our method so that virtually any given protein space could be data mined, in search of antimicrobial peptides.

**Conclusion:**

The results show that the Fourier transform based method with the property based coding strategy could be used to scan the peptide space for discovering new potential antimicrobial peptides.

## Background

### Introduction

Antimicrobial peptides (AMPs) have been discovered as the first line of defense in almost all major groups of organisms like animals, plants and microbes [1,2]. As an integral part of the innate immune system, they also help in immunological boosting, against harmful pathogens. Several similar AMPs have been identified from different organisms, proving their evolutionary significance in the defense mechanism [3]. These AMPs could serve as safe and efficient drug candidates against the microbes that become resistant to synthetic drugs. They offer a broad spectrum of activity against diverse microbial systems, like bacteria, fungi, viruses and protozoa [4]. Several modes of actions of these AMPs have been suggested and identified, like pore forming, inhibition of cell-wall/nucleic acid/protein synthesis etc [5]. As with the modes of action, they also show a diverse function, in addition to the primary antimicrobial activity, such as tissue repair and tissue-remodelling process etc [6]. Many AMPs have also been identified with potential anticancer activities.

The significance of the study on AMPs is justified from their prominent role in the host defense mechanism of human beings. Researchers have identified AMPs in several parts of the body including skin, alimentary canal, urogenital, respiratory, gastrointestinal, mammary, ocular, hematopoietic and lymphoid systems [7,8]. They have been implicated in several diseases such as psoriasis, dermatitis, cystic fibrosis and inflammatory bowel diseases [9]. A rat homologue of human antimicrobial peptide Cathlicidin has been shown to play a critical role in the innate immunity of the central nervous system. Cathlicidin has also been detected in sufficient quantity in human and bovine milk [10]. In recent years, AMPs have been implicated in several unexplained human inflammatory disorders. This has led to the search for novel therapeutic approaches.

### Antimicrobial peptide properties

Currently there are about a thousand AMPs identified and databased [11,12]. Broadly divided as those with or without disulphide bridges, they are usually cationic and possess amphipathic stereo geometry. In the Antimicrobial Peptide Database [11], the AMPs are also grouped based on their activity, as antibacterial, antifungal, antiviral and anticancer peptides. While many of the properties such as charge (cationic) and hydrophobicity are similar, AMPs also have group specific properties such as highest Cysteine (Cys) content in antiviral, and lowest Cys content in anticancer AMPs. In general AMPs are small in size, typically <10 kDa, with less than 50 amino acid residues. The majority of them have a net positive charge (cationic) in the range of +2 to +7 at pH 7. The Glycine content is high in almost all the AMPs while Methionine is the least found amino acid. Sequence and structural motifs have also been identified in AMPs [13].

### Discovering new AMPs

Ever since the potential use of AMPs as natural antibiotics has been envisaged, a lot of research has gone in to discovering and designing new AMPs for improved activity and efficiency. Synthetic peptide variants of the known AMPs have been synthesized in the laboratories and tested for their activity [14]. The synthetic analogues to natural AMPs are mostly generated by mutational experiments, like single amino acid substitution [15,16]. Rational design of novel AMPs, by the structural modification of the natural ones has yielded good results [17-20]. Several de novo design methods have also been shown to construct artificial AMPs with improved activity, enhanced specificity and therapeutic index [21-24]. Peptide engineering based on the biophysical studies is also being attempted, to create novel AMPs [25]. A new field of study, peptidomics has come to help researchers who are in search of identifying new peptides [26]. The APD (Antimicrobial Peptide Database) offers an interface to predict the antimicrobial activity of any submitted peptide sequences, based on a simple residue analysis and count method.

Even though many new AMPs with improved activity have been reported, seldom has any method been used to tap the potential of huge amount of genomic and proteomic data, to discover hitherto unknown AMPs. As of mid 2005, data is available to the public from 266 complete genomes, 1,226 ongoing genome projects [27], 184,304 experimentally verified protein sequences [28], 1,779,481 translated protein sequences [29], 44,202,133 reported nucleotide sequences [30] and 31,217 protein structures [31]. This is the molecular space available for screening for potential natural antimicrobial peptides.

We have developed a Fourier transform based screening method that could mine the rich source of data available in the genomic and proteomic databases, and identify potential AMPs. The parallel version of our method could also virtually scan the entire peptide space for any given length, for the presence of AMPs. Using our established method, we are currently working on to virtually mine the whole human peptide space for the potential antimicrobial peptides.

### Proteins and Fourier Transformation

Fourier transform (FT) has been extensively used to study periodicities in introns, genes, repeats, coding and non-coding regions in DNA sequences [32-35]. In proteins, Fourier transform methods have been used for classification and other structure based studies such as analysis of symmetry and repeating structural units or patterns, prediction of secondary/tertiary structure prediction, prediction of hydrophobic core, motifs, conserved domains, prediction of membrane proteins, prediction of cellular localization etc [36-40]. FT methods have also been successfully used to study the phylogenetic relationships between genetic sequences [41]. Most of the research in protein science, using Fourier transform methods has focused on structural aspects. The most often used methods to code biological sequences in to numerical sequences, for signal processing, are the binary indicator sequence method for DNA sequences, and the EIIP index and hydropathy index [42,43] method for protein sequences. Other index methods have also been tested for protein sequence coding. In this study we propose a high performance, protein property based function prediction method using Fourier transform. We have shown that the use of a combination of distinct protein property based indices, in relation to its function, gives the best representation of the peptides function in the frequency domain. We have successfully implemented and tested our method for the prediction of antimicrobial activity.

In this paper we address 3 main areas, property based protein coding method, antimicrobial activity prediction using Fourier transforms and a parallel environment to data mine both naturally occurring and artificial peptide space (virtually generated random sequences).

## Results and discussion

**Figure 1:**
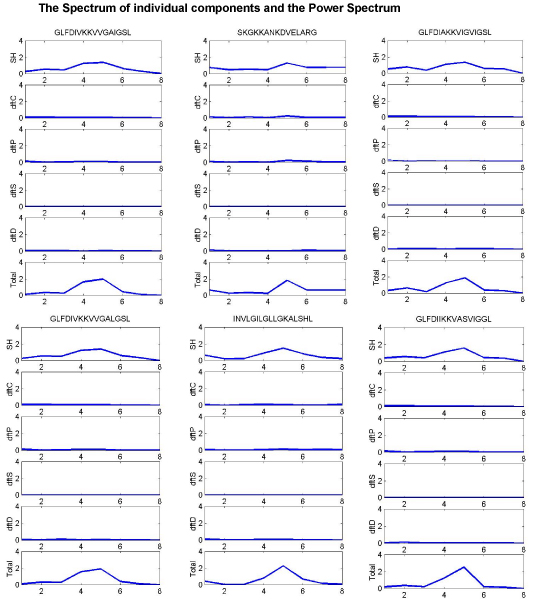
**Property based coding method**. The spectrum of the individual components (SH-hydrophobicity, dftC-charge, dftP-polarity, dftS-cysteine, dftD-amino acid distribution) and the power spectrum (Total) of 6 different Antimicrobial sequences. The Power spectrum (Total) shows a clear peak at period 5 in all the 6 sequences, which is more distinct, sharp and high in magnitude, compared to the same peak in dftH. The noise level is also highly minimized in the Total spectrum, compared to the dftH. The Total spectrum seems to represent the comprehensive nature of all the properties taken in to consideration.

The spectrum of each one of the indices Eq. (1–5) and their representation as a whole Eq. (8) are plotted in Figure 1, where we could clearly visualize the effect of the holistic approach (the combined spectrum represented by all the contributing properties), in terms of a distinct peak at period 5. In Figure 1, even though we could observe the peak, in the individual plot of the spectrum obtained only with hydrophobicity index, the magnitude of the peak is higher and distinct in the total power spectrum. The Signal to noise ratio at the period 5 is distinctly higher in the total power spectrum compared to that of the SNR at 5 of the hydrophobicity spectrum. A clear boosting of the antimicrobial activity signal at period 5 and a great reduction in the noise level are the two important outcomes that we observe, as a result of the holistic approach. The individual Fourier spectrum of other indices sometimes showed the peak at period 5, but failed to show up other times.

The Euclidean metric to screen the sequences based on the similarity to the reference spectra was identified as D = 3. The signal to noise ratio was also identified as >5 in period 4|5|6.

Mining the 10,000 random sequences yielded 3 positive hits, predicted as containing antimicrobial activity. The predicted peptide sequences are:

Hit1: AQQAQSRREVTHMVQH

Hit2:CIVYCMEIGAIRRCAK

Hit3:FFPEMREYARDCEQSP

**Figure 2:**
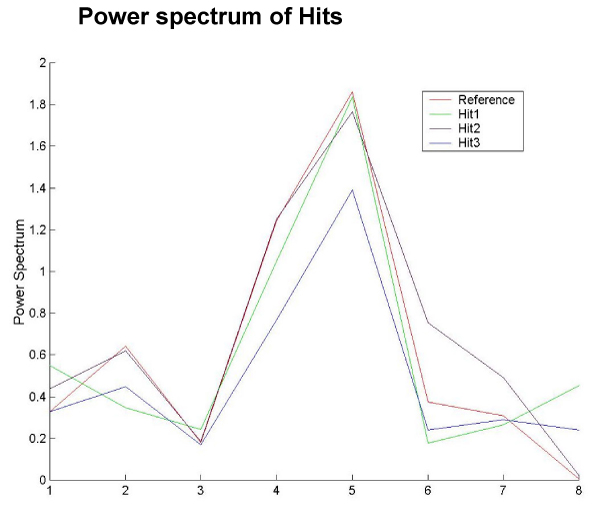
**Power spectrum of new hits**. Plot of the reference power spectrum and the power spectrum of the three hits. A distinct peak is clearly seen at period 5.

All the three hits show a clear and distinct peak at period 5 (Figure 2).

For Hit 1 and Hit 2 APD's simple prediction tool predicted that they might form alpha helices and might have at least 3|6 residues on the same hydrophobic surface, might interact with membranes and has a chance to be antimicrobial peptides. But for the Hit 3, APD's prediction said that the sequence has negative charge and has very little chance to be an antimicrobial peptide. The sequence analysis details for the above predictions by APD are given in the Table 1.

The BLAST similarity search results for all the 3 hits against APD is given in the Table 2, with similarity scores in percentage.

**Figure 3:**
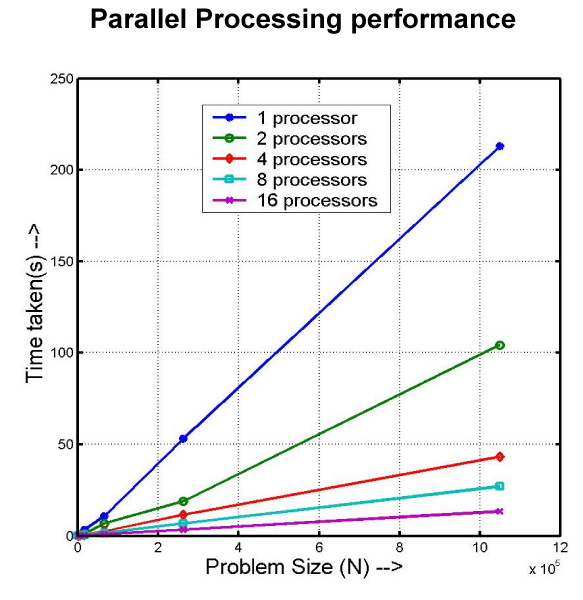
**Parallel Processing performance**. Plot of computation time VS. problem size for different number of processing elements, during the virtual data mining of 16^5 ^random sequences.

The Parallel scan for predicting the peptides with antimicrobial activity among the 16^5 ^random sequences gave a huge reduction in the computing time, as the number of processors was increased, as shown in the Figure 3. All our 3 hits predicted under serial scan were also among the 12 hits obtained in the parallel run.

## Conclusion

In this work, we have established a method based on Fourier transformation, using property-based sequence coding approach, to predict antimicrobial activity of peptide sequences. Fourier analysis of antimicrobial peptides using the holistic approach yields the boosted signal at period 5, the noise level is reduced and the signal to noise ratio also increases compared to the Fourier spectrum of the individual amino acid indices, particularly in comparison with the hydrophobicity indexed spectrum. As an initial step, we derived at a Euclidean metric threshold and Signal to Noise Ratio threshold level, based on the analysis of a particular class of antimicrobial peptide (anticancer peptides). To test our method, we mined the randomly generated 10,000 peptide sequences of 16 residue length, using the above threshold value, in their respective frequency domain. This gave 3 positive hits. Analysis of these potential antimicrobial peptide sequences (positive hits), in comparison to the only available simple prediction tool, validated our predictions. Out of the 10,000 random peptides that we screened, many of them had helical structures and hydrophobic patterns (tested using the online tool ) but failed to pass through our filter. This shows that antimicrobial property is determined by the complex "multidimensional signatures" rather than by individual properties, as suggested by Yount and Yeaman [13]. The 10000 random peptides did contain many amphipathic, or membrane-spanning peptides. But those peptides that did not have significant AMP activity were filtered out, as indicated by the period 5 behaviour. The remaining hits were examined individually and were found to have significant AMP property. It is possible that some false negatives might have occurred. At least among the 10000 random peptides that were screened there were no false positives. We also showed the use of a parallel version of our program in data mining huge peptide space, for potential antimicrobial activity. We are currently fine-tuning our method to include all the different classes of antimicrobial peptides and also to classify the hits based on corresponding classes.

Our future work is aimed towards virtually generating and data mining the whole peptide space for the antimicrobial peptides. We are also working on the sequence input method to mine, the available proteomic and genomic data in the public databases.

## Methods

### Datasets

All the different groups of peptide sequences used in the analyses presented were retrieved from the Antimicrobial Peptide Database. These include individual anticancer peptide sequences taken for a specific study, and the sixteen residue anticancer peptide sequences used to generate a consensus sequence .

### Property based protein coding

The concept of structure-activity relationship is well established in the field of cheminformatics, where techniques like Quantitative Structure Activity Relationship (QSAR) are used for predicting the biological activity of chemical compounds. We propose a similar method for predicting the functions of the proteins, based on their physicochemical properties. In this approach, we coded the antimicrobial peptide sequences, with numerical indices based on their physicochemical properties in order to represent the function – antimicrobial activity. Most of the Fourier transform methods in protein science have used either hydrophobicity index or EIIP (electron-ion interaction potential) index. In our proposed property based function prediction method, we used 5 different indices to represent each peptide sequence, viz. hydrophobicity index (x_h _[n]), charge index (x_c _[n]), polarity index (x_p _[n]), cysteine index (x_s _[n]) and aminoacid distribution index (x_d _[n]). X_h _[n] was coded using Kyte-Doolittle scale [5], x_c _[n] was coded based on the charge of the aminoacid, +1 if positive, -1 if negative and 0 if neutral. X_p _[n] was coded based on the polarity of the aminoacids, +1 for polar residues and -1 for non-polar residues, x_s _[n] was coded as +1 for Cysteine and 0 for the rest of the residues and x_d _[n] was coded based on the APD statistics of the residue distribution among various AMPs.









*x*_*h*_[*n*]={Kyte-Doolittlehydrophobicityscale (5)

In formulae 1–5,*k *stands for any of the 20 amino acids.

### Fourier transforms

The antimicrobial peptide sequence is denoted as x [n] of length N. This peptide sequence is first decomposed into its 5 component indicator sequences x_α _[n] (α = c, p, s, d, h) as per our property based coding method. The DFT of every indicator sequence x_α _[n] is given by



where the Power Spectrum of the antimicrobial peptide x [n] with the DFT X_α _[n] is computed as:



The initial coding was done in Matlab.

The spectra of the individual indicator sequences were analyzed along with the power spectrum.

### Metric

The initial analyzes involved several representative peptides from all the four classes of AMPs (anticancer, antiviral, antifungal and antibacterial), to identify the presence of distinct signals that would be characteristic of the antimicrobial activity. We also studied the contribution of the individual properties (different aminoacid indices), to the nature of the antimicrobial activity.

With that initial analysis, we further went on to study only a group of AMPs classified as anticancer peptides in APD. This involved analysis of 18 anticancer peptides in their frequency domain. Among these we took the power spectrum of all those peptides with 16 residue length (APD ID 34556, 34556, 2222, 3355, 2211, 2334), to obtain a reference spectrum RPS.



where ns=number of 16aa sequences averaged, PS is the power spectrum of the individual 16 aa sequences.

The relative difference of each one of the original 18 anticancer peptide spectrum to that of the common reference spectrum was measured in terms of Euclidean metric. The power spectrum of all the 16 residue AMPs were averaged for the purpose of generating a representative spectra. The Euclidean distance between peptide power spectrums PS1 and PS2 of length *l *is given by



The signal to noise ratio (SNR) was also computed for all the individual 16 residue AMPs and compared with the representative power spectra. The SNR is computed using the average spectrum :



where 

Detailed algorithm could be found in paper [32].

### Data mining the peptide space

To test the performance of our method, we generated 10000 random peptide sequences of 16 residue length, using Perl scripts. For every sequence in the multiple sequence file, the indicator sequences are generated based on our proposed property based coding method, Fourier transforms obtained and power spectrum calculated. The power spectrum of every sequence was analyzed based on signal to noise ratio as well as Euclidean metric with the threshold value of 3. The sequences that contain the high SNR at period 4/5/6 and D = 3, are captured as potential antimicrobial peptides.

Every positive hit from the data mining was analyzed through APD tools, to cross check the prediction accuracy. These hits obtained from the random peptide sequences were submitted to APD's simple antimicrobial activity prediction tool. The positive hits were also used to perform BLAST similarity searches against the APD.

### Parallel environment

Since the virtual peptide space is huge, to the order of 20^16 ^(we only used 10000 random sequences to test our method using the serial Matlab code) for the 16 residue peptides, added to the 5 indicator sequences the number for the 16 residue peptide space goes to 5 × 20^16^. The FFT (Fast Fourier Transformation) computation complexity is *O(N log N) *where *N *is the problem size for the aforementioned order of number of sequences and this would require a huge amount of computing time.

A theoretical calculation of computing time for executing the serial program would be:

T_s_=(5*t*_*c*_*l*log*l*+5*lt*_*c*_)N = 5*t*_*c*_*lN*(1+log*l*) (11)

where T_s _= time taken for each sequence × total number of sequences, t_c_=the time taken for each multiply-add operation, t_s_=the startup time and *l *is the sequence length.

Under a parallel environment, if the problem is equally partitioned among p processors, the theoretical run time Tp would be:



Thus to circumvent the problem of huge computing time running the data mining application in a single processor machine, and to utilize the advantage of parallel processing, we developed our method to perform the screening in a high performance environment. This would enable the virtual screening of any given peptide space. The parallel version was implemented in C and MPI. The program was test executed on the SGI supercomputer with 128 processors, at the Mississippi Center for Supercomputing Research (MCSR), using PBS (portable batch system) scripts. We parallely screened 16^5 ^randomly generated 16 residue peptides for predicting those with the antimicrobial activity.

## Authors' contributions

VN developed and implemented the algorithm, prepared the datasets, and drafted the manuscript along with NK. SL provided assistance in the math part. BM, CZ and MOE directed the computational, parallel computing and biology part respectively. YD directed the whole research and critically revised the manuscript. All authors read and approved the final manuscript.

## Figures and Tables

**Table 1:** Sequence analysis details for APD predictions

	G|P (#)	Naa (#)	Paa (#)	HR (%)	NetC	BI (kcal/mol)
hit1	0	1	4	31	3	3.75
hit2	1	1	3	62	2	0.79
hit3	2	4	2	31	-2	-

G|P is the number of Glycine|Proline residues, Naa is the number of negatively charged aminoacids, Paa is the number of positively charged aminoacids, HR is the hydrophobicity ratio, NetC is the net charge and BI is the Boman Index in kcal/mol.

**Table 2:** BLAST similarity search results against APD

	Similar hits	% similarity
hit1	AP00402	30
	AP00403	25
	AP00163	24.13
hit2	AP00511	38.88
	AP00214	38.09
	AP00303	35.29
hit3	AP00335	31.57
	AP00288	26.08
	AP00072	25.92

G|P is the number of Glycine|Proline residues, Naa is the number of negatively charged aminoacids, Paa is the number of positively charged aminoacids, HR is the hydrophobicity ratio, NetC is the net charge and BI is the Boman Index in kcal/mol.
